# Hypertension Assessment via ECG and PPG Signals: An Evaluation Using MIMIC Database

**DOI:** 10.3390/diagnostics8030065

**Published:** 2018-09-10

**Authors:** Yongbo Liang, Zhencheng Chen, Rabab Ward, Mohamed Elgendi

**Affiliations:** 1 School of Electrical Engineering, Guilin University of Electronic Technology, Guilin 541004, China; liangyongbo001@gmail.com (Y.L.); chenzhcheng@163.com (Z.C.); 2 School of Electrical and Computer Engineering, University of British Columbia, Vancouver, BC V6T 1Z4, Canada; rababw@ece.ubc.ca; 3 Department of Obstetrics & Gynecology, University of British Columbia, Vancouver, BC V6Z 2K8, Canada; 4 BC Children′s & Women′s Hospital, Vancouver, BC V6H 3N1, Canada

**Keywords:** pulse oximeter, blood pressure monitoring, pulse arrival time, global health, digital medicine, wearable devices

## Abstract

Cardiovascular diseases (CVDs) have become the biggest threat to human health, and they are accelerated by hypertension. The best way to avoid the many complications of CVDs is to manage and prevent hypertension at an early stage. However, there are no symptoms at all for most types of hypertension, especially for prehypertension. The awareness and control rates of hypertension are extremely low. In this study, a novel hypertension management method based on arterial wave propagation theory and photoplethysmography (PPG) morphological theory was researched to explore the physiological changes in different blood pressure (BP) levels. Pulse Arrival Time (PAT) and photoplethysmogram (PPG) features were extracted from electrocardiogram (ECG) and PPG signals to represent the arterial wave propagation theory and PPG morphological theory, respectively. Three feature sets, one containing PAT only, one containing PPG features only, and one containing both PAT and PPG features, were used to classify the different BP categories, defined as normotension, prehypertension, and hypertension. PPG features were shown to classify BP categories more accurately than PAT. Furthermore, PAT and PPG combined features improved the BP classification performance. The F1 scores to classify normotension versus prehypertension reached 84.34%, the scores for normotension versus hypertension reached 94.84%, and the scores for normotension plus prehypertension versus hypertension reached 88.49%. This indicates that the simultaneous collection of ECG and PPG signals could detect hypertension.

## 1. Introduction

Hypertension is a major factor of many cardiovascular diseases (CVDs), which are a group of disorders of the heart and blood vessels, including coronary heart disease, cerebrovascular disease, peripheral arterial disease, rheumatic heart disease, etc. [1]. Although sometimes there are symptoms of headache, lack of breath, chest pain, and so on, for most people with hypertension, there are no symptoms at all. Therefore, it is also known as the “silent killer”, and 13% of global death is attributed to it [1]. With each heartbeat, blood is pumped via the contraction of the heart and flows through the whole body following the arterial system. Blood pressure is formed by the main propulsion of the heart’s pumped blood and blockage of the microcirculatory system. Therefore, the higher is the blood pressure, the more difficult it is for the heart to pump. This undoubtedly increases the burden of the heart and, in the long term, will lead to a series of CVDs and damage to the heart, blood vessels, brain, kidneys, and so on.

Fortunately, blood pressure is the most important preventable factor of CVDs. Early prevention and management of hypertension are the major and most effective means of improving people’s health levels worldwide. Healthy lifestyles (healthy diet, non-alcohol consumption, non-tobacco use, and physical activity), early detection, evaluation of blood pressure levels, proper diagnosis, and treatment with low-cost medication are beneficial in the prevention and control of hypertension [2]. The seventh report of the Joint National Committee on Prevention, Detection, Evaluation, and Treatment of High Blood Pressure (JNC7) [3], which is funded and published by the US National Institutes of Health, is widely adopted. According to this report, different BP levels are divided into different hypertension categories, including normotension, prehypertension, stage 1 hypertension, and stage 2 hypertension. Due to the number of research participants, this study adopted the three BP categories of normotension, prehypertension, and hypertension, labeled according to the BP ranges of the JNC7 report [3].

Clearly, earlier attention and treatment are more effective in preventing hypertension and other CVDs. However, as we know, most hypertension patients have no symptoms in the stage of elevated blood pressure and even in hypertension. Thus, many people miss the best time for treatment and experience some complications. However, some physiological signals change based on blood pressure level [4,5], such as electrocardiogram (ECG) and photoplethysmography (PPG). The morphological changes in physiological signals mainly reflect the change of function status of the heart and vascular system. Therefore, the morphological information of PPG could be used to assess hypertension [6]. For this purpose, the Medical Information Mart for Intensive Care (MIMIC) database [7,8] was used to collect the dataset for this study, which involves arterial blood pressure (ABP), ECG and PPG signals.

Many researchers have used the MIMIC database assuming that all simultaneously collected signals were synchronized [9,10,11,12,13]. However, the creators of the MIMIC database have reported errors in the data matching and alignment in some recordings, as mentioned by Clifford et al. [14], confirming that not all signals were synchronized. This contradiction motivated our study, and we thought it would be useful to test the synchronicity-dependent features (features that rely on the time interval between ECG and PPG events) and asynchronicity-dependent features (features that rely only on features extracted from PPG events) to gain insights about the usability of the MIMIC database for evaluating hypertension either by using ECG and PPG signals or by using PPG alone.

The rest of this paper is organized as follows: Section 2 explains the methods used in this study, including data collection, signal process, and feature extraction. Section 3 shows the comparison results of the different classification models and different feature sets. Finally, Section 4 and Section 5 discuss the results and conclusions on the differences and optimizations of arterial wave propagation theory and PPG morphological theory, respectively.

## 2. Methods

### 2.1. Database

In this study, the data were collected from the MIMIC database, which is a free-to-use database that contains tens of thousands of Intensive Care Unit (ICU) patients [7,8]. The recordings with arterial blood pressure (ABP, measured using a catheter in the radial artery), electrocardiograph (ECG) and photoplethysmography (PPG) were collected and archived for this study. During data collection, there were some abnormal and noisy recordings, for example, missing peak, pulsus bisferiens, no signal (sensor-off), and so on. These recordings were excluded in this study. Meanwhile, to explore and model the relationship between ABP, ECG, and PPG signals, 120 one-second-length signals with stable, complete ECG, ABP, and PPG signals without heart disease except for hypertension were cut from raw recordings for each subject. In the end, 121 subjects’ records, each 120 s in length, were collected in this research.

### 2.2. PAT Feature

The definition of PAT in the literature is highly inconsistent [15]. On many occasions, PAT is used to refer to Pulse Transit Time (PTT), and in other publications, PTT referred to as PAT [15]. Moreover, the calculation of PAT is not consistent in the literature, but some general convention exists. It is worthy to note that the R-wave of an ECG and the foot of the PPG waveform is perhaps the most commonly used in the literature. In 2013, Choi et al. [16] tested three different measurement points for PAT, including the peak (PAT_R*S*_), middle (PAT_R*b*-2_ or PAT*_W_*_-1_), and end (PAT_RO_) of the PPG waveform. Their study recommended the use of PAT-middle as it is highly correlated with BP, and therefore, in our study we used the PAT_R*b*-2_ to represent the PAT feature.

### 2.3. PPG Features

As the referenced blood pressure source of this study, the original ABP signal did not undergo any preprocessing. Systolic blood pressure (SBP) and blood pressure categories were extracted and labeled directly from the original ABP waveform signal [17]. A 0.5–10 Hz 4th Chebyshev II bandpass filter was adopted to remove the noise of raw PPG signals and improve the signal quality index (SQI) [18], and a 0.5–40 Hz 4th Butterworth bandpass filter was used to filter the noise of raw ECG signals [19]. Additionally, a normalization process was conducted for filtered ECG and PPG signals to divide the pulsating part of blood volume (the AC part) by the non-pulsating part (the DC part). Further, two forward differential processes were implemented to acquire the velocity waveform of PPG signals (VPG) and the acceleration waveform of PPG signals (APG). Note, the first order differential to the original PPG signal to obtain the VPG signal, and the second order differential to the original PPG signal to obtain the acceleration of PPG waveform signals. To visualize main events within these signals, the TERMA framework [20] and Eventogram [21] can be used. Finally, ECG, PPG, VPG, and APG were together regarded as the feature extraction signal resources.

**Figure 1:**
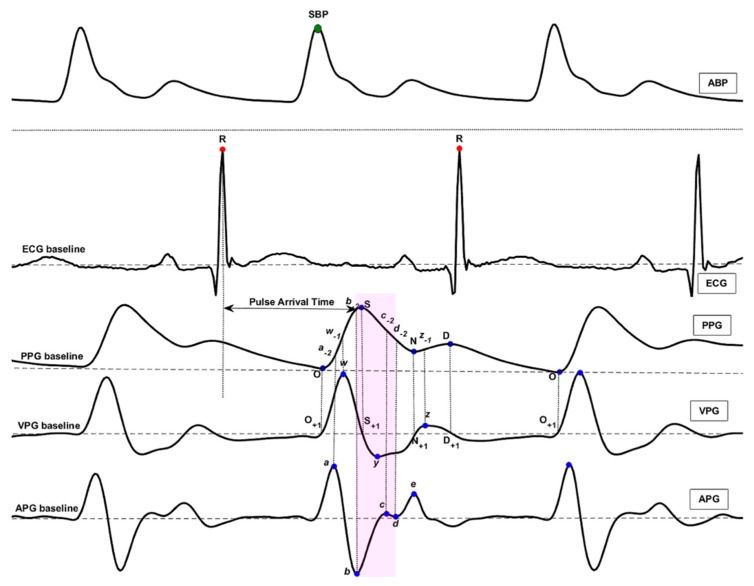
The characteristics of arterial blood pressure (ABP), electrocardiogram (ECG), photoplethysmogram (PPG), velocity photoplethysmogram (VPG), and acceleration photoplethysmogram (APG) waveforms. The definition of feature points can be found in our past research [25]. The PPG amplitude is represented by the feature name and the amplitudes is represented by the height from PPG baseline to feature points such as *a*, *a*_-1_, *a*_-2_, etc. The shaded area contains features associated with hypertension.

In this study, the feature points were extracted beat by beat, and the heart-beat pair was divided by the R wave of the ECG, which was identified by a reliable detector [22,23,24]. In one beat period, some feature points of PPG and its derivatives were defined [25], and the detailed waveforms and names are clearly marked in Figure 1. Several types of features were defined based on ECG, PPG, VPG, and APG signals. They are the pulse arrival time (PAT) extracted from ECG and PPG signals; PPG morphology features (135 features) extracted from PPG, VPG, and APG signals, including time spans (23 features); PPG amplitudes (14 features); features of VPG and APG (20 features); waveform area (4 features); power area (15 features); ratio (43 features); and slope (16 features). The detailed features are defined as follows:

**Time Span (23):** The time span features are expressed as their letters with a dash on top (e.g., $\bar{SD}$).
**Features of PPG Amplitude (14):** The S, N, D, *w*_-1_, *a*_-2_, *b*_-2_, *c*_-2_, etc. features were defined in PPG waveform. They represent the amplitude of the corresponding waveform from the PPG baseline.
**Features of VPG and APG (20):** The *a*, *b*, *c*, *d*, and *e* features were defined in the APG waveform, and the *w*, *x*, *y*, and *z* features were defined in the VPG waveform. They represent the amplitude of the corresponding waveform from the APG baseline and VPG baseline. Other features based on these features were also defined, such as *b/a*, *c/a*, *d/a*, *e/a*, *(b-c-d-e)/a*, *(b-c-d)/a*, and so on.
**Waveform Area (4)**: The waveform AC component area features are expressed as their letters with a polyline on top (e.g., $\hat{\mathrm{OS}}$).
**Power Area (15)**: The power area features are expressed as their letters with a brace on top (e.g., $\overbrace{\mathrm{OS}}$). For example, the $\overbrace{\mathrm{OS}}$ feature represents the quadratic sum of the curve point from the onset point O to the systolic peak S.
**Ratio (43)**: The ratio features are expressed directly as their ratio formulae (e.g., $\bar{OS}/\bar{OO}$, $\hat{OS}/\hat{OO}$).
**Slope (16):** The slope features are expressed as their letters with a tilde on top (e.g., $\tilde{\mathrm{OS}}$).

### 2.4. Classification Models

Several classifiers are discussed in the literature; however, we selected four distinctive classifiers: Logistic Regression, AdaBoost Tree, Bagged Tree, and K Nearest Neighbors (KNN). These classifiers represent different classification theories such as regression, decision tree, cluster, and bagged decision tree. From the results, we can see that KNN achieves better classification performance than the others. As we know, KNN is a very common classifier that can be used in many applications and can be easy to realize.

In this study, the dataset was divided into a training set (70%) and a testing set (30%). In the training phase, the training adopted 10-fold cross validation to validate the generalization ability of the trained model. The trained model was then used to predict for the testing set. The F1 score was calculated as an evaluation measure, as follows:

(1) F1 score=2×Recall×Precision/(Recall+Precision)

where Precision = TP/(TP + FP) and Recall = TP/(TP + FN). TP stands for true positives, FP stands for false positives, and FN stands for false negatives. To comprehensively evaluate the trained models, various evaluation indexes were used, including sensitivity (SE), specificity (SP), and the F1 score, which is the harmonic mean of precision and sensitivity. In this study, all the signal processing, modeling, and evaluating were carried out via MATLAB software (R2017b version), developed and released by MathWorks (Natick, MA, USA) company.

## 3. Results

In our past research [18,25,26], we conducted a BP management study based on a clinical dataset collected in China by a PPG device designed for that study. In that study, 10 PPG features were evaluated and selected for BP category classification. Based on that study, the same 10 PPG features and new extracted PAT features were adopted here to classify the different BP categories to optimize the arterial wave propagation theory and PPG morphological theory. The 10 PPG features are shown in Table 1. We can see that the features are mainly in the *bd* segment.

The 10 PPG features and PAT feature were used to classify the different BP categories, which include normotension (46 subjects), prehypertension (41 subjects), and hypertension (34 subjects). Meanwhile, four different classifiers were trained and tested. Table 2 shows the classification performances of the different trials and feature sets. In general, the KNNs achieved the best classification performance compared to the other classifiers. Our findings were that the PPG features were more beneficial in classifying BP categories than the single PAT feature. Further, the combination of PAT feature and PPG features greatly improved the classification performance of using only PPG features.

A comparison was also carried out with our past research. Because of the difference in the PPG datasets of this study (MIMIC database) and the past one (collected by a designed device [26]), the 10 PPG features were also used to classify the BP categories to compare with the past research, and, further, an optimization using the PAT feature and 10 PPG features was compared. To our knowledge, no study has previously investigated this research question on the same database.

## 4. Discussion

PPG signal is affected by heart activity, vascular wall function, and peripheral arterial status [27]. Therefore, it is a very complex physiological signal with abundant information [28,29]. The morphological information of PPG signals plays an important role in the analysis of cardiovascular activity. In past research, many PPG morphological features [29,30] have been proposed, including the Crest Time, Delta T, Augmentation Index, Large Artery Stiffness Index [31], PPG intensity ratio [32], etc. Some novel features showed excellent performance in BP prediction or hypertension management. However, most of the research was conducted based on a small quantity of healthy participants [33]. A more comprehensive and systematic study needs to be implemented to improve and validate the arterial wave propagation and PPG morphological theories.

Several issues have been studied in our past research, such as optimal SQI [34], optimal filter for PPG signal [18], detection of PPG morphological characteristics [35,36,37,38], generating diagnostic PPG features for abnormality evaluation [25], compressing PPG signals [39], and so on. To continue in our previous research direction, we aimed in this study to: (1) identify special signatures in both PAT feature and PPG features for hypertensive and prehypertensive subjects and to differentiate them from normotensive subjects; and (2) use such features to monitor management of BP level and to check treatment compliance using the MIMIC database.

PAT and PPG features reflect different physiological information: PAT can indicate the transmission of the arterial wave in the blood vessel, while PPG features can indicate the status change of vascular tissue and blood volume. Therefore, three experimental analyses were implemented to determine the feature differences in the different BP level classifications (normotension versus prehypertension, normotension versus hypertension, and normotension plus prehypertension versus prehypertension). Based on our past research, 10 PPG features were used in this study for these experimental classifications. Table 2 shows the 10 PPG features that were evaluated in our past research. To determine the characteristics of features to classify, four different type classifiers were adopted: the AdaBoost Tree, Logistic Regression, K-Nearest Neighbors (KNN), and Bagged Tree. The KNN classifier showed the best performance compared with the other models.

PAT has some limitations as it cannot classify these three categories of blood pressure levels; PPG features showed better performance in classifying hypertension from normotension than the other experiments. Furthermore, the feature set of PAT feature and 10 PPG features obviously improved the classification performance for all three experiments. This indicates that the combination of arterial wave propagation theory and PPG morphological theory can be beneficial in modeling and quantizing the BP formation, which is comprehensive and complex. Various influencing factors work together to determine and affect blood pressure, such as a heart′s cyclical activity, vasomotion, total blood volume, cardiac output, vascular elasticity, peripheral resistance, and so on. Therefore, the blood pressure level is the physiological response of the cardiovascular system, and cardiac function, total blood volume, and vascular elasticity play decisive roles in the formation of blood pressure. Hence, it is feasible to use arterial wave propagation theory to explain blood transmission and to use PPG morphological theory to explain the changes of vascular aging, stiffness, and compliance that generally occur at different BP levels.

In our past research, the PPG signal was collected as 1000 Hz sample frequency and 12 bits ADC, and the blood pressure was collected by a commercial BP device: the Omron 7201 BP device [26]. Comparing the results of this study to the past study, we saw that using the PPG feature set scored similar results but was lower in accuracy than the past research. The MIMIC database used in this study contains a wealth of physiological and pathological information and waveform records to study and explore physiological models and algorithms. However, more attention should be paid to this database. MIMIC data were collected from ICU wards, which means that many of the participants may have received medication or other medical treatment that may lead to BP abnormalities. In addition, it is very likely that the age of most of the participants is generally high. As we know, PPG signal is a complex physiological signal; therefore, the low quality of raw PPG signals makes it challenging to extract physiological characteristics correctly.

The accurate identification of feature points is very important, especially based on the PPG morphology method, and the PPG signal quality is the key. Because the sampling frequency is only 125 Hz in the MIMIC database, this could lead to the identification error of each characteristic point. Therefore, this actually limits the database from being extended to blood pressure research, especially based on PPG morphology, to achieve the dynamic monitoring of blood pressure. Moreover, many recordings have ECG, ABP (invasive, from one of the radial arteries), and PPG (named “PLETH”) in the MIMIC database. However, collecting satisfactory recordings with ECG, ABP, and PPG simultaneously [33] is not easy for many reasons, such as various heart diseases and abnormal or missing signals.

In addition, the ABP signal is a continuous invasive blood pressure signal collected using a catheter. Thus, there is a little difference between the dataset in this study and our past research, which collected the blood pressure using an Omron 7201 cuff BP device [26]. Even so, the result of this study is similar to but just a bit lower than the past. This indicates that it is feasible to use the PPG morphological features to manage BP levels. Fortunately, the feature set with PAT feature and PPG features significantly improved the BP classification performance. This emphasizes the importance of arterial wave propagation theory in BP formation.

Note, it is assumed that the linear relationship between BP and PAT calculated from the MIMIC database are inconsistent from subject to subject. If all signals were synchronized, perhaps the correlation would be more salient. However, there is an overall trend of correlation between BP and PAT in the recordings used from the MIMIC database.

The proposed method could play a significant role in the early detection of hypertension in low- and middle-income countries (LMICs). Note that an estimated 1.04 billion people had hypertension in LMICs in 2010 [40]. Having a non-invasive method that relies on ECG and PPG signals, which follows the framework recommended in Ref. [41] for tackling noncommunicable diseases by achieving simplicity and reliability, may decrease morbidity and mortality rates, especially for those living in LMICs.

## 5. Conclusions

PPG morphological features were shown to achieve better classification performance than PAT using the MIMIC database. PPG signals contain sufficient physiological information about the activity of the heart and arteries. Although they are easily affected by many factors, the 10 evaluated PPG features achieved an acceptable classification performance. This indicates that the PPG signal, which is the status response of the heart and arteries, varies according to the BP levels, such as normotension, prehypertension, and hypertension. Interestingly, adding the PAT feature to the PPG feature set improved the overall classification performance, even though not all ECG and PPG signals in the MIMIC database were synchronized. Our results show that the PAT feature and PPG features have great potential to manage BP levels.

## Figures and Tables

**Table 1:** Photoplethysmogram (PPG) morphological features. ANOVA stands for analysis of variance.

Feature #	PPG Features	Normotension	Prehypertension	Hypertension	ANOVA *p*-Value
1	$\overbrace{S_{+1}c_{-1}}/\overbrace{O_{+1}O_{+1}}$	0.52 ± 0.45	0.52 ± 0.42	0.38 ± 0.45	<0.01
2	$\tilde{b_{-2}d_{-2}}$	−2.94 ± 7.66	−3.35 ± 7.11	−0.93 ± 8.65	<0.01
3	$\bar{Sc_{-2}}$	0.06 ± 0.05	0.06 ± 0.04	0.04 ± 0.05	<0.01
4	*c*_-__2_ /S	0.79 ± 0.17	0.78 ± 0.16	0.83 ± 0.19	<0.01
5	$\bar{Sd_{-2}}$	0.09 ± 0.06	0.09 ± 0.04	0.09 ± 0.06	<0.01
6	*(b-c-d)/a*	−0.53 ± 0.64	−0.61 ± 0.59	−0.48 ± 0.61	<0.01
7	*d*	−0.52 ± 0.61	−0.41 ± 0.57	−0.69 ± 0.63	<0.01
8	*c_-_*_1_ */w*	−0.25 ± 0.27	−0.26 ± 0.26	−0.14 ± 0.28	<0.01
9	*d/a*	−0.21 ± 0.26	−0.17 ± 0.25	−0.27 ± 0.25	<0.01
10	$\tilde{Sc_{-2}}$	−6.79 ± 6.03	−7.34 ± 5.71	−5.31 ± 7.01	<0.01

**Table 2:** Classification performance of PAT and PPG features. In this table, SE, SP, and F1 represent the sensitivity, specificity, and F1 score, respectively. Normal, Prehyp. and Hyp. represent normotension, prehypertension, and hypertension, respectively. The results of this table were achieved based on the test set.

Trial	Feature Set	AdaBoost Tree			Logistic Regression			K-Nearest Neighbors			Bagged Tree		
		SE (%)	SP (%)	F1 (%)	SE (%)	SP (%)	F1 (%)	SE (%)	SP (%)	F1 (%)	SE (%)	SP (%)	F1 (%)
**Normal (46)** **vs.** **Prehyp. (41)**	PAT feature	67.42	65.46	66.88	56.91	56.27	56.85	46.69	73.29	53.93	67.63	65.24	66.95
	10 PPG features	90.13	41.81	72.76	71.65	46.01	63.66	79.48	77.07	78.62	79.20	77.14	78.48
	(PAT + 10 PPG) features	75.67	72.72	74.67	67.35	56.20	63.92	83.92	84.76	84.34	83.50	84.26	83.88
**Normal (46)** **vs.** **Hyp. (34)**	PAT feature	63.48	80.56	68.10	63.04	80.71	67.85	40.09	93.08	54.08	63.48	80.56	68.10
	10 PPG features	75.65	88.81	80.11	62.09	82.47	67.94	84.78	91.31	86.94	81.65	91.09	84.98
	(PAT + 10 PPG) features	87.57	94.33	90.15	78.87	82.62	79.11	94.26	96.17	94.84	92.70	96.39	94.13
**(Norm + Prehyp.) (87)** **vs.** **Hyp. (34)**	PAT feature	40.44	95.37	53.19	45.51	88.76	52.38	40.27	95.37	53.01	40.44	95.37	53.19
	10 PPG features	53.16	94.63	63.79	35.02	94.55	47.10	74.40	93.92	78.44	68.09	94.94	75.32
	(PAT + 10 PPG) features	74.22	95.23	79.71	55.20	91.20	62.26	87.47	95.93	88.49	85.87	96.50	88.22
